# Ameliorative Activity of Ethanolic Extract of *Artocarpus heterophyllus* Stem Bark on Alloxan-induced Diabetic Rats

**DOI:** 10.15171/apb.2018.017

**Published:** 2018-03-18

**Authors:** Basiru Olaitan Ajiboye, Oluwafemi Adeleke Ojo, Oluwatosin Adeyonu, Oluwatosin Imiere, Babatunji Emmanuel Oyinloye, Oluwafemi Ogunmodede

**Affiliations:** ^1^Department of Chemical Sciences, Biochemistry Programme, Afe Babalola University, Ado-Ekiti, Ekiti State, Nigeria.; ^2^Department of Chemical Sciences, Industrial Chemistry Programme, Afe Babalola University, Ado-Ekiti, Ekiti State, Nigeria.

**Keywords:** Artocarpus heterophyllus stem bark, Alloxan, Serum lipid profiles, Haematological parameters

## Abstract

***Purpose:*** Diabetes mellitus is one of the major endocrine disorders, characterized by impaired insulin action and deficiency. Traditionally, Artocarpus heterophyllus stem bark has been reputably used in the management of diabetes mellitus and its complications. The present study evaluates the ameliorative activity of ethanol extract of Artocarpus heterophyllus stem bark in alloxan-induced diabetic rats.

***Methods:*** Diabetes mellitus was induced by single intraperitoneal injection of 150 mg/kg body weight of alloxan and the animals were orally administered with 50, 100 and 150 mg/kg body weight ethanol extract of Artocarpus heterophyllus stem bark once daily for 21 days.

***Results:*** At the end of the intervention, diabetic control rats showed significant (p<0.05) weight reduction, abnormal haematological parameters, high serum lipids (except high density lipoprotein) concentrations, increased creatinine, bilirubin and urea levels with decreased in albumin level when compared with non-diabetic control rats. All these alterations were reverted to normal after administered with different doses of ethanol extract of Artocarpus heterophyllus stem bark most especially at 150 mg/kg body weight which exhibited no significant (p>0.05) different with non-diabetic rats.

***Conclusion:*** The results suggest that ethanol extract of Artocarpus heterophyllus stem bark may be useful in ameliorating complications associated with diabetes mellitus patients.

## Introduction

Diabetes mellitus is a chronic metabolic disorder characterized by derangements in carbohydrate, protein and lipid metabolisms, due to defective or deficiency in insulin secretion and action.^1^ Insulin is a hormone secreted by the beta cells of the Islets of Langerhans of the pancreas, it helps in glucose uptake by the cells, thereby prevents increase in fasting blood glucose levels.^2^

Diabetes mellitus is also associated with hyperglycaemia, which promotes oxidative stress through non-enzymatic glycation and glucose auto-oxidation.^3^ This oxidative stress has been implicated in the complications of diabetes mellitus, including dyslipidaemia, a major risk factor for cardiovascular disease,^4^ anaemia, nephropathy, and hepatopathy.^3^ It is believed that therapeutic agents (like metformin, gliberclamide etc.) normally reduces the effects of oxidative damages in diabetes mellitus patients and therefore ameliorate its complications.^3^ But these drugs are characterized with some side effects such as hypoglycaemia, hypersensitivity, gastrointestinal disturbances, lactic acidosis, liver toxicity amongst others.^5,6^

Furthermore, in recent years, the search for alternative therapeutic agents in the management/treatment of diabetes has been the major focus of scientific researches across the globe.^7^ Added to this, the therapeutic efficacy of medicinal plant due to their believed minimal side effects and its affordability over modern synthetic drugs has been recommended. In this regards many herbal medicines and medicinal plants^7^ have been used traditionally for the control and management/ treatment of diabetes mellitus in different parts of the world.^8^ It has been reported that screening of medicinal plants for therapeutic purposes, is an important aspect in drug development because they may possess anti-aneamia, anti-dyslipideamia, anti-nephropathy and anti-hepatopathy which may be useful in the management of diabetes mellitus.^9^

*Artocarpus heterophyllus* (jack fruit) is an example of plant that may be used in this regards, it belongs to a family of *Moraceae* and grown in tropical climates. *Artocarpus heterophyllus* has been considered a rich source of carbohydrates, minerals, dietary fiber and vitamins amongst others.^10^ Its stem bark has also been reported to be of great importance in the management of diabetes mellitus locally.^11^ In addition, the inhibitory ability of *Artocarpus heterophyllus* stem bark on alpha-amylase and alpha-glucosidase has been documented.^12^

Therefore, the present study was designed to examine the ameliorative effects of ethanol extract of *Artocarpus heterophyllus* stem bark on haematological parameters, serum lipid profiles, liver and kidney functions indices of alloxan-induced diabetic rats.

## Materials and Methods

### Chemicals

Alloxan used was a product of Sigma Aldrich (St. Louis, MO, USA). Glibenclamide used was a product of Sandoz SA (Pty) Ltd. (Gauteng, South Africa). All assay kits used were obtained from Randox while other chemicals used were purchased from Merck Chemical (Germany).

### Collection of Plant Material 

The fresh peeled stem bark of *Artocarpus heterophyllus* were collected at a farm in Ibadan, Oyo State, Nigeria. This was then identified and authenticated at the Department of Plant Science, Ekiti State University, Ado-Ekiti, Nigeria.

### Extract preparation 

The stem bark of *Artocarpus heterophyllus* was shade-dried to a constant weight. Thereafter, kitchen blender was used to blend the dried stem bark of *Artocarpus heterophyllus* into fine power and stored in air-tight containers. Thereafter, 100 grams of powdered plant sample was extracted with 1 litre of 70% ethanol for 48 hours. The extract was then filtered with Whatman filter paper and the filtrate was evaporated to dryness using a freeze dryer. The extract (SBAH) was reconstituted in distilled water and used for subsequent analysis.

### Qualitative phytochemical screening 

The methods described by^13-15^ were employed in these determinations. These include:

- Test for Tannins
1 ml of SBAH was boiled with 20 ml of distilled water in a test tube and filtered. Thereafter, a few drops of 0.1% ferric chloride was added and formation of a green or a blue–black coloration confirm the presence of tannin
- Test for Phlobatannins:
Deposition of a red precipitate when 2 ml of SBAH was boiled with 1% aqueous hydrochloric acid was taken as evidence for the presence of phlobatannins.
- Test for Saponin:
5 ml of the SBAH was boiled with 20 ml of distilled water in a water bath and filtered. Then 10 ml of the filtrate was mixed with another 5 ml of distilled water and shaken vigorously till formation of a stable persistent froth. The frothing was further mixed with 3 drops of olive oil and shaken vigorously until formation of emulsion which confirms the presence of saponin.
- Test of Flavonoids:
In this test, 3 ml of 1 % aluminium chloride was added to 5 ml of SBAH. A yellow coloration was observed indicating the presence of flavonoids.
- Test for Steroids
2 ml of acetic anhydride was added to 2 ml SBAH, thereafter, 2 ml H_2_SO_4_. The formation of blue or green indicate the presence of steroids.
- Test for Terpenoids
5 ml of SBAH was mixed with 2 ml of chloroform, then 3 ml concentrated H_2_SO_4_ was added to form a layer. The formation of reddish brown coloration at the interface indicate the presence of terpenoids.
- Test for Cardiac Glycosides
5 ml of SBAH was added to 2 ml of glacial acetic acid containing one drop of ferric chloride solution. Thereafter 1 ml of concentrated sulphuric acid was added. Formation of a violet-green ring appearing below the brown ring, in the acetic acid layer, indicates the presence of glycoside.
- Test for Alkaloids:
1 ml of the SBAH was stirred with 5 ml of 1% aqueous HCl on a steam bath, then filtered while hot. Distilled water was added to the residue and 1 ml of the filtrate was added with a few drops of either Mayer’s reagent (Potassium mercuric iodide- solution) or Wagner’s reagent (solution of iodine in Potassium iodide) or Dragendorff’s reagent (solution of Potassium bismuth iodide). The formation of a cream colour with Mayer’s reagent and reddish-brown precipitate with Wagner’s and Dragendorff’s reagent confirm the presence of alkaloids.
- Test for Anthraquinone
5 ml of SBAH was mixed with 10 ml of benzene, filtered and 5 ml of 10 % NH_3_ solution was added to the filtrate. Thereafter, the mixture was shaken until the appearance of pink, red or violet colour.
- Test for Chalcones
2 ml of ammonia solution was added to 5 ml of SBAH and formation of a reddish colour confirmed presence of chalcones.
- Test for Phenol
5 ml of the SBAH was pipetted into a 30 ml test tube, then 10 ml of distilled water was added. 2 ml of ammonium hydroxide solution and 5 ml of concentrated amyl alcohol were then added to the mixture and left for 30 minutes. Formation of bluish green colour indicate the presence of phenol.

### Experimental Animals 

A total of 36 albino rats’ weighting between 180-200 g obtained from Animal Holding Unit of Afe Babalola University, Ado-Ekiti, Ekiti State, Nigeria were used in this study. The animals were kept under standard environmental conditions for 21 days of the experimental period. Prior to this the animals were acclimatized for 7 days. All the animals had free access to food and water throughout the experimental period. All procedures followed were in accordance with the ethical standards of Afe Babalola University Animal Committee with Ethical Approval Number (ABUAD/SCI/004). Also the principle of Laboratory Animal Care were followed throughout the experimental period.

### Induction of Diabetes 

Freshly prepared alloxan monohydrate of 150 mg/kg body weight dissolved in 0.9% sterile NaCl of pH 7^16^ was administered intraperitoneally to rats in group B to F to induce diabetes. Prior to this, their fasting blood glucose levels had been determined. Also, after 48 hours of alloxan induction, the rats fasting blood glucose levels were assessed with the aid of Acucheck Advantage II glucometer and those that had fasting blood glucose level ≥ 200 mg/dl were considered diabetic and used for the study.^17^ The rats were divided into six groups with six rats per group as follows:

Group A: Non-diabetic control rats received distilled water (3 mg/kg body weight)

Group B: Diabetic-control rats received distilled water (3 ml/kg body weight)

Group C: Diabetic rats received glibenclamide (5 mg/kg bodyweight)

Group D: Diabetic rats received 50 mg/kg body weight of SBAH

Group E: Diabetic rats received 100 mg/kg body weight of SBAH

Group F: Diabetic rats received 150 mg/kg body weight of SBAH

### Collection and analysis of samples 

On the 20th day of oral administration, the animals were fasted overnight for 12 hours and on the 21st day the animals were anaesthetized with halothane and then sacrificed. The animal’s blood were then collected by cardiac puncture into ETDA bottles and plain sample bottles. The former were used for haematological parameters determination while the latter were allowed to clot for two hours and centrifuged at 3000 rpm for 10 minutes, after which serum was recovered for analysis.

### Haematological parameters determination 

The levels of packed cell volume (PCV), haemoglobin (HB), white blood cell (WBC), red blood cell (RBC), neutrophil (N), lymphocyte (L), monocyte (M), eosinophil (E), mean corpuscular haemoglobin concentration (MCHC), mean corpuscular haemoglobin (MCH) and mean corpuscular haemoglobin (MCH) were determined using automated haematology analyzer (System KX-21N^Tm^, Japan).

### Serum lipid profile determinations 

The method^18^ modified by^19^ was employed for determination of cholesterol concentration. The method of^20^ was employed for determination of triglycerides concentration and high density lipoprotein (HDL) concentration, while the Friedwald equation^21^ was used for determining very low density lipoprotein (VLDL) concentration and low density lipoprotein (LDL) concentration. Atherogenic index (AI) and coronary artery risk index (CRI) were carried out using^22^ and^23^ respectively.

### Determination of some liver and kidney function indices

The method of^24^ was used for albumin determination, while the method described by^25^ was employed for bilirubin determination. Creatinine was determined using the method described by Tietz and co-workers^26^while urea was estimated according to the method of Fawcett and Scott.^27^ Alanine and aspartate aminotransferase (ALT and AST) activities were determined using the method of Reitman and Frankel.^28^ Alkaline phosphatase was determined as described by Wright et al.^29^

### Statistical Analysis 

The data were analysed with students’ T-test and one way ANOVA. Values of p<0.05 were considered significant.

## Results and Discussion

Ajiboye *et al*^30^ reported that plants were endowed with series of secondary metabolites like terpenoids, phenolic, lignins, stilbenes, tannins, flavonoids, quinones, coumarins, alkaloids, amines, betalains amongst others. They were free radical scavenging, making them serve as antioxidant compounds and possess antidiabetic activity amongst others. In this study, the ethanol extract of *Artocarpus heterophyllus* stem bark demonstrated the presence of saponins (Table 1) which possess cholesterol and fasting blood glucose levels lowering effect making it useful for diabetes mellitus patients.^31^

**Table 1:** Qualitative screening of some phytochemical constituents of ethanol extract of *Artocarpus heterophyllus* stem bark

**Parameters**	***Artocarpus heterophyllus stem bark***
Tannin	+
Phlobatannin	+++
Saponin	+++
Flavonoid	+
Steroid	-
Terpenoid	++
Cardiac glycosides	+++
Alkaloid	+++
Anthraquinone	-
Chalcones	-
Phenol	+++

+++ = much abundant, ++ = less abundant, + = minute and - = absent

The flavonoid (Table 1) in the ethanol extract of *Artocarpus heterophyllus* stem bark could make it useful as antidiabetic and anticancer agents.^32^ Also, Ajiboye *et al*^30^ reported the role of flavonoid in preventing oxidation of LDL therefore reducing the risk for the development of atherosclerosis, one of diabetes mellitus complication. The presence of tannin could make the extract useful in hastening wound healing in diabetes mellitus patients.^33^ The tannin, phenol and alkaloid in Table 1 also strengthen the possibility of antidiabetic activity of the extract.^33,34^

**Figure 1:**
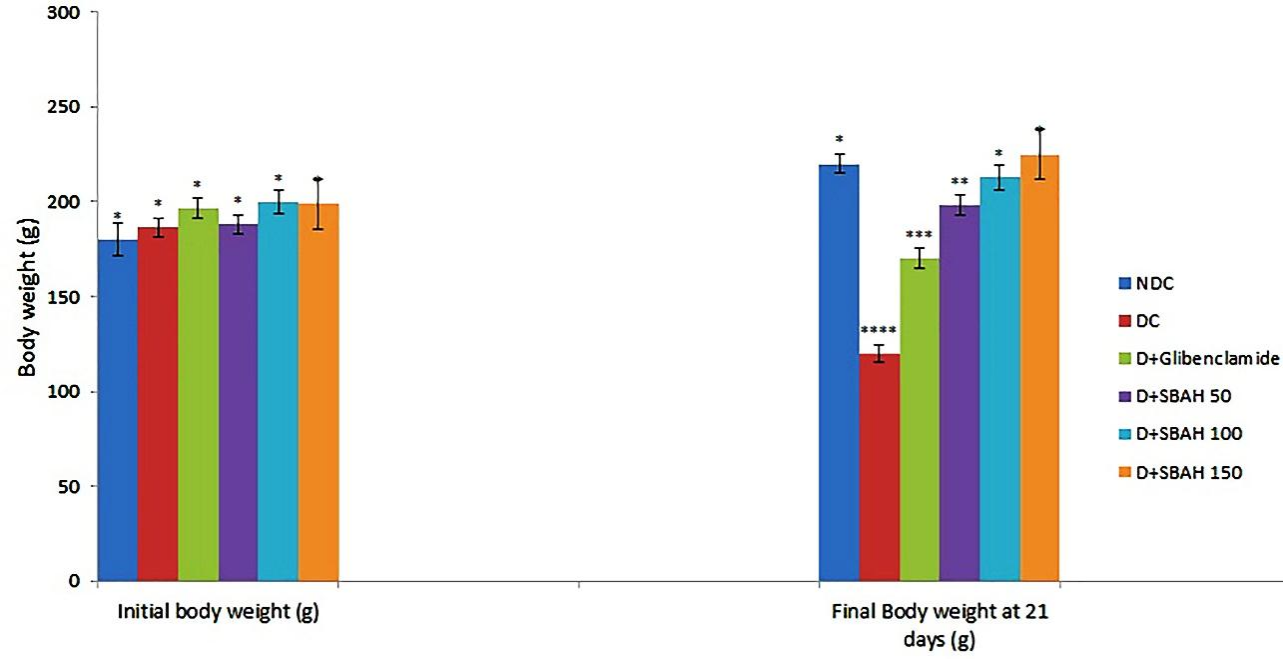
Effect of ethanol extract of *Artocarpus heterophyllus* stem bark on body weight of alloxan-induced diabetic rats
Bars with different superscript are significantly different at p<0.05
NDC (non-diabetic control), DC (Diabetic control), D+ SBAH 50 (Diabetic rats received 50 mg/kg body weight of *Artocarpus heterophyllus* stem bark), D + SBAH 100 (Diabetic rats received 100 mg/kg body weight of *Artocarpus heterophyllus* stem bark, and SBAH 150 (Diabetic rats received 150 mg/kg body weight of *Artocarpus heterophyllus* stem bark

Alloxan is one the chemicals that are widely used to induce diabetes in experimental animals due to its toxicity on the beta cell of pancreas islet, leading to weight reduction, anaemia, hyperlipidaemia, hepatopathy and nephropathy.^35^ In this study there were significant (p<0.05) weight reductions in diabetic control rats when compared to non-diabetic control rats. Administration of diabetic rats with 50, 100 and 150 mg/kg body weight of *Artocarpus heterophyllus* stem bark significantly (p<0.05) increased the body weight especially at 150 mg/kg body weight (Figure 1). This is in accordance with^36^ that diabetes mellitus patients were characterized by loss of body weight due to increased protein catabolism, as a result of insulin insufficiency.

Diabetes mellitus has been characterized by anaemia as reported by,^37^ which is supported by the present study especially in diabetic control rats (Table 2). This might due to excess glucose in the hemoglobin, results in glycosylated hemoglobin with decrease in red blood cell (RBC) and packed cell volume (PCV), an indicator of imbalance between its synthesis and destruction.^38^ Anaemia in diabetes mellitus patients may also be attributed to damage in the synthesis of erythropoietin, hormone whose function in promoting formation of the red blood cells, probably due to excess blood glucose.^38,39^ This was coupled with significant (p<0.05) decrease in MCV, MCHC and MCH

**Table 2:** Effect of ethanol extract of *Artocarpus heterophyllus* stem bark on haematological parameters of alloxan-induced diabetic rats

**Parameters**	**NDC**	**DC**	**D+glibenclamide**	**D+ SBAH50**	**D+ SBAH100**	**D+150 SBAH**
**PCV (%)**	46.48±0.24*	19.26±1.24****	38.16±1.40***	37.10±0.09***	42.10±0.05**	46.20±1.10*
**HB (g/dl)**	18.42±0.05*	8.42±0.10*****	10.21±1.15****	12.40±0.01***	15.10±1.10**	18.20±1.10*
**WBC (x 10** ^ 3 ^ **/μl)**	3.10±1.00*	1.01±0.02***	2.80±0.03**	2.94±0.01**	3.08±0.08*	3.20±0.05*
**N (%)**	63.01±0.10*	42.19±1.10*****	52.10±1.20****	54.10±1.10***	59.10±0.20**	62.94±1.10*
**L (%)**	42.23±0.16*	26.10±1.01*****	30.10±0.02****	33.42±0.10***	38.44±1.16**	42.96±0.08*
**M (%)**	11.20±0.03*	6.01±0.06****	8.20±1.04**	9.60±0.89**	10.01±0.05**	11.46±0.14*
**E (%)**	5.10±0.01*	1.28±0.12****	3.10±0.06***	3.98±0.06***	4.22±0.10*,**	4.98±0.94*
**RBC (x10** ^ 11 ^ **/l)**	5.94±0.10*	2.48±0.04****	3.28±0.13***	3.84±0.48***	4.46±0.10**	5.68±0.14*
**MCHC (g/dl)**	42.10±0.04*	28.40±1.10*****	32.18±0.08****	36.40±1.20***	39.20±0.08**	42.84±0.10*
**MCV (fl)**	96.42±0.10*	58.29±0.56****	86.30±0.94***	92.40±1.02**	93.82±0.94**	96.10±0.04*
**MCH (pg)**	40.44±1.16*	30.01±0.46****	34.21±0.14***	34.96±0.12***	38.41±0.12**	40.80±0.08*

Each value is a mean of six replicates ± SEM. Values with different superscript along the row are significantly different at p<0.05. PCV (packed cell volume), HB (haemoglobin), WBC (white blood cell), N (neutrophil), L (lymphocyte), M (monocyte), E (eosinophil), RBC (red blood cell), MCHC (mean corpuscular haemoglobin concentration), MCV (mean corpuscular volume), MCH (mean corpuscular haemoglobin), NDC (non-diabetic control), DC (Diabetic control), D+ SBAH 50 (Diabetic rats received 50 mg/kg body weight of *Artocarpus heterophyllus* stem bark), D + SBAH 100 (Diabetic rats received 100 mg/kg body weight of *Artocarpus heterophyllus* stem bark, and SBAH 150 (Diabetic rats received 150 mg/kg body weight of *Artocarpus heterophyllus* stem bark

In addition, the significant (p<0.05) reduction observed in WBC and its differentials (lymphocytes, neutrophil, monocytes and eosinophil) (Table 2) in diabetes control rats might suggest decrease in immune cells in mopping up free radicals generated.^40^ Treatment of diabetic rats with ethanol extract of *Artocarpus heterophyllus* stem bark reversed all this haematological abnormalities especially at 150 mg/kg body weight probably due antioxidant nature of the plant.

There were significant (p<0.05) increase in serum lipid profiles and calculated atherogenic and coronary risk indices with a reduction in serum high density lipoprotein-cholesterol (HDL) in diabetic control rats when compared to non-diabetic rats (Table 3). This is in accordance with Wilson and Islam^41^ that hyperlipidaemia is another factor directly linked to diabetes mellitus, also, high circulating blood lipid concentrations secrete humoral factors like resistin and adiponectin that alter insulin sensitivity. In this study, hyperlipidaemia were encouraged in diabetic control rats by increasing the levels of low density lipoprotein-cholesterol (LDL), very low density lipoprotein-cholesterol (VLDL) and total cholesterol.^42,43^ Oral intervention of diabetic rats administered with 50, 100 and 150 mg/kg body weight of ethanol extract of *Artocarpus heterophyllus* stem bark as well as glibenclamide demonstrated reduction in atherogenesis and coronary artery diseases likewise augmenting the HDL-cholesterol. This is in line with the report of amongst others.^43^

**Table 3:** Effect of ethanol extract of *Artocarpus heterophyllus* stem bark on serum lipid Profiles, atherogenic and coronary indices of alloxan-induced diabetic rats

**Groups**	**TC (mg/dl)**	**TG (mg/dl)**	**LDL (mg/dl)**	**VLDL (mg/dl)**	**HDL (mg/dl)**	**AI**	**CRI**
**NDC**	80.40 ± 0.50*	64.10 ± 1.20*	23.37 ± 0.01*	12.82 ± 1.03*	44.21 ± 0.05*	0.82 ± 0.10*	1.82 ± 0.01*
**DC**	110.40±0.40*****	120.10±0.10*****	66.26±0.40*****	24.02±0.05***	20.12±0.18*****	4.49±0.40*****	5.49±0.04****
**D+glib**	92.10 ± 1.20****	84.20 ± 0.11****	43.16±1.20****	16.84±0.84**	32.10±1.10****	1.87±0.24****	2.87±0.32***
**D+ SBAH 50**	90.10 ±0.40***	76.20±1.10***	38.62±1.12***	15.24±1.40*,**	36.24±1.40***	1.49±0.02***	2.49±0.14***
**D+ SBAH100**	84.48 ±0.42**	68.10±1.12**	29.88±0.08**	13.21±1.01*	40.98±1.01**	1.06±0.02**	2.06±0.06**
**D+ SBAH150**	81.20±0.12*	63.89±0.14*	24.48±0.80*	12.78±1.06*	43.94±1.20*	0.85±0.05*	1.85±0.02*

Each value is a mean of six replicates ± SEM. Values with different superscript across the column are significantly different at p<0.05. D+Glib (D+glibenclamide), TC (total cholesterol), TG (Triglyceride), LDL (low density lipoprotein-cholesterol), VLDL (very low density lipoprotein-cholesterol), HDL (high density lipoprotein-cholesterol), AI (atherogenic index), CRI (coronary risk index), NDC (non-diabetic control), DC (Diabetic control), D+ SBAH 50 (Diabetic rats received 50 mg/kg body weight of *Artocarpus heterophyllus* stem bark), D + SBAH 100 (Diabetic rats received 100 mg/kg body weight of *Artocarpus heterophyllus* stem bark, and SBAH 150 (Diabetic rats received 150 mg/kg body weight of *Artocarpus heterophyllus* stem bark

Moreover, diabetes mellitus has been characterized by hepatopathy and nephropathy,^44^ which is supported by the present study (Table 4). This is buttressed with high level of liver-function enzymes like AST, ALT and ALP and other biochemical parameters (urea, creatinine, albumin and bilirubin) in diabetic control rats compared to others groups. The hepato and nephro protective abilities of 50, 100 and 150 mg/kg body weight of ethanol extract of *Artocarpus heterophyllus* stem bark supported by reduction in serum AST, ALT and ALP activities, as well as urea, creatinine (breakdown of muscle mass, as result of increase in gluconeogenesis) and bilirubin levels with increase in albumin (probably due to decrease in gluconeogenesis) levels when compared to diabetic control rats.

**Table 4:** Effect of ethanol extract of *Artocarpus heterophyllus* stem bark on serum ALT, AST, ALP and other biochemical parameters of alloxan-induced diabetic rats

**Groups**	**ALT (u/l)**	**AST (u/l)**	**ALP (u/l)**	**Albumin (g/l)**	**Bilirubin(mg/dl)**	**Creatinine(mg/dl)**	**Urea (mg/dl)**
**NC**	72.02±1.20*	84.10±0.06*	143.02±1.20*	20.23±1.20*	4.20±0.12*	2.64±0.42**	34.20±1.10*
**NDC**	126.20±0.90****	140.11±1.30****	500.11±1.02*****	10.10±1.12*****	15.40±0.10****	6.20±1.01***	86.19±0.18*****
**D+glib**	84.20±1.20***	96.10±0.06***	320.14±0.11****	13.79±0.60****	8.20±1.30***	3.20±0.10**	56.24±1.12****
**D+SBAH 50**	83.94±1.42***	95.01±2.01***	284.01±0.42***	16.21±0.27***	6.38±0.12***	2.90±0.08*	50.12±1.10***
**D+SBAH100**	76.20±1.02**	88.10±1.10**	246.15±0.12**	18.53±0.80**	5.80±0.19**	2.70±0.06*	41.12±0.18**
**D+SBAH150**	73.40±0.41*	85.25±0.20*	153.21±2.12*	20.10±0.31*	4.64±0.18*	2.66±0.10*	34.69±1.14*

Each value is a mean of six replicates ± SEM. Values with different superscript across the column are significantly different at p<0.05. NDC (non-diabetic control), DC (Diabetic control), D+glib (D+glibenclamide), D+ SBAH 50 (Diabetic rats received 50 mg/kg body weight of *Artocarpus heterophyllus* stem bark), D + SBAH 100 (Diabetic rats received 100 mg/kg body weight of *Artocarpus heterophyllus* stem bark, and SBAH 150 (Diabetic rats received 150 mg/kg body weight of *Artocarpus heterophyllus* stem bark

## Conclusion

The results of the present study show that various doses of ethanol extract of *Artocarpus heterophyllus* stem bark demonstrates the presence of different phytochemical compounds which serve as antioxidant, therefore improved weight gain, ameliorate anaemia, hyperlipidaemia to normolipidaemia, and demonstrated hepato and nephro-protective in diabetic rats.

## Acknowledgments

The authors are very grateful to all the Technologists in Biochemistry Laboratory of Afe Babalola University, Ado-Ekiti.

## Ethical Issues

The Ethical Committee of the Afe Babalola University approved this study with approval number ABUAD/SCI/004.The study was performed in accordance with the rules of the established Animal Ethical Committee of Afe Babalola University, Ado-Ekiti.

## Conflict of Interest

The authors declare no conflict of interest
